# Comparison of Refractive Status and Anterior Segment Parameters of Juvenile Open-Angle Glaucoma and Normal Subjects

**DOI:** 10.4274/tjo.68915

**Published:** 2018-12-27

**Authors:** Ufuk Elgin, Emine Şen, Murat Uzel, Pelin Yılmazbaş

**Affiliations:** 1 University of Health Sciences, Ulucanlar Eye Research Hospital, Opthalmology Clinic, Ankara, Turkey; 2 Sandıklı State Hospital, Opthalmology Clinic, Afyon, Turkey

**Keywords:** Juvenile glaucoma, optical biometry, anterior segment parameters, axial length, spherical equivalent

## Abstract

**Objectives:**

Our aim was to compare the refractive status and anterior segment parameters of patients with juvenile open-angle glaucoma (JOAG) and normal subjects.

**Materials and Methods:**

Twenty-five recently diagnosed cases of JOAG and 24 normal subjects were included in this prospective controlled clinical trial. Central corneal thickness (CCT), anterior chamber depth (ACD), lens thickness (LT), axial length (AL), K1 and K2 keratometry, and white-to-white distance (WTW) measurements were performed with optical biometry (LenStar LS 900, Haag Streit Diagnostics). Spherical equivalent (SE) values and anterior segment parameters were statistically compared by chi-square, Kolmogorov- Smirnov, and independent samples t-tests.

**Results:**

The mean age of the 15 male and 10 female JOAG patients was 11.8±2.78 (8-18) years and the mean age of the 14 male and 10 female normal subjects was 11.58±3.04 (7-16) years (age: p=0.51; sex: p=0.18). Mean intraocular pressure in the JOAG group before treatment was 30.08±4.3 mmHg. The mean SE values of the JOAG and the control group were -1.94±1.86 (+2.35/-5.5) and -0.76±2.03 (+2.25/-4.85) diopters, respectively (p=0.048). JOAG patients had lower mean CCT values (p=0.016) and higher mean AL and ACD values (p=0.049 and p=0.016). There were no significant differences between the groups for LT, WTW, K1, or K2 (p=0.61; p=0.52; p=0.95; p=0.31 respectively).

**Conclusion:**

JOAG patients were found to be more myopic and have lower CCT and greater AL and ACD values than normal subjects. These anterior segment changes may be associated with myopia, which is common in JOAG.

## Introduction

Juvenile open-angle glaucoma (JOAG) is a more aggressive subtype of primary open-angle glaucoma (POAG), with an age at diagnosis between 5 and 35 years.^1,2^ It is mostly a hereditary disease and has autosomal dominant inheritance.^3,4^ JOAG is known to be associated with higher intraocular pressure (IOP) levels and fluctuations than POAG.^1,2,5,6^ Myopic refractive state and male gender have also been reported to be associated with JOAG.^2^

Noncontact biometers can provide some anterior segment measurements using the low-coherence reflectometry method.^7,8^ Central corneal thickness (CCT), anterior chamber depth (ACD), lens thickness (LT), axial length (AL), K1 and K2 keratometry, and white-to-white distance (WTW) can be measured by optical biometers with the help of diode laser.^7,8^

Because myopia was reported to be associated with JOAG, some differences in anterior segment parameters and AL are expected in these patients. In our study, we aimed to compare refractive status and anterior segment parameters in patients with JOAG and normal subjects.

## Materials and Methods

This prospective controlled clinical trial included 25 eyes of 25 patients recently diagnosed with JOAG and 24 eyes of 24 normal subjects. The glaucoma patients were examined between December 2015 and December 2017 in the Glaucoma Department of Ulucanlar Eye Research Hospital and the control subjects were recruited from among similarly aged patients who presented to our hospital for routine ophthalmological examination. Our study was approved by the Ethics Committee of Ankara Numune Training Hospital and written informed consent was obtained from the patients’ parents.

All patients underwent detailed ophthalmologic examinations including best-corrected visual acuity with Snellen chart, anterior and posterior segment examinations, and intraocular pressure (IOP) measurements with Goldmann applanation tonometer. In addition, central corneal thickness measurements by ultrasonic pachymeter, visual field examinations with Humphrey automated perimeter (*Humphrey* Field Analyzer; SITA Standard 24-2 strategy, model *750i*; Zeiss-*Humphrey* Instruments, Dublin, CA), gonioscopic examination by Goldmann 3-mirror lens in *cooperative* patients, and retinal nerve fiber layer (RNFL) analysis by spectral-domain optical coherence tomography (Spectralis OCT; Heidelberg Engineering, Heidelberg, Germany) were also done for the diagnosis of glaucoma. For patients who were not cooperative enough for gonioscopy, the iridocorneal angle was visualized by Scheimpflug imaging system (Pentacam, Oculus, Lynwood, WA).

Diagnosis criteria for JOAG were optic nerve head changes such as cup-to-disc (C/D) ratio ≥0.3 and localized neuroretinal rim defects, IOP ≥22 mmHg, glaucomatous changes of optic disc and retinal nerve fiber layer in OCT analysis, and visual field defects such as nasal step, Seidel, or arcuate scotoma, and abnormal glaucoma hemifield test in *cooperative *patients. Patients older than 18 years old and those with history of any systemic diseases were excluded from the study, as well as any eyes with history of keratitis, uveitis, congenital ocular disease, contact lens use, and ocular surgery or trauma. In addition, eyes with best-corrected visual acuity worse than 20/30 and high *spherical equivalent (SE) values* (<-6.0 D or >+3.0 D) were excluded from the study.

### Statistical Analysis

CCT, ACD, LT, AL, K1 and K2 keratometry, and WTW measurements were obtained by optical biometer (Haag-Streit LenStar^®^ LS 900 Optical Biometer Switzerland) by the same experienced physician (M.U.) for glaucoma cases and control subjects. The measurements were done before anti-glaucoma treatment in glaucoma cases and also before cycloplegia. Chi-square, Kolmogorov-Smirnov, and independent samples t-tests were used for statistical analysis. The Kolmogorov-Smirnov test was used for testing normal distribution of the data and independent samples t-test was used for comparison of the data. The eyes with higher initial IOP values were included in the glaucoma group. The right eyes were included in the control group.

## Results

The mean age of the 15 male (60%) and 10 female (40%) patients with JOAG was 11.8±2.78 (8-18) years and the mean age of the 14 male (58.3%) and 10 female (41.7%) normal subjects was 11.58±3.04 (7-16) years. Differences in age and sex distribution between the groups were not statistically significant (age: p=0.51; sex: p=0.18). Ten (40%) of our JOAG patients had family history of JOAG (Table 1).

The mean IOP before treatment was 30.08±4.3 (22-38) mmHg and the mean IOP in the control group was 16.2±2.4 (10-19) mmHg (p<0.001) (Table 1). On clinical examination, vertical C/D ratio was 0.3-0.5 in 9 eyes, 0.6-0.7 in 13 eyes, and 0.8-0.9 in 3 eyes. The mean SE values of the JOAG and the control group were -1.94±1.86 (+2.35 to -5.5) and -0.76±2.03 (+2.25 to -4.85) diopters, respectively (p=0.048) (Table 1). Reliable visual field results could be obtained for 20 JOAG patients. The mean value of the mean deviation of these 20 eyes was -9.61±4.23 dB (between -4.5 and -18.23 dB). Mean circumpapillary RNFL thickness of the 25 eyes with glaucoma was 76.83±12.6 (60.03-100.02) µm.

JOAG patients had significantly smaller mean CCT values (p=0.016) and larger mean AL and ACD (p=0.049 and p=0.016). There were no significant differences between the groups for LT, WTW, K1 or K2 (p=0.61; p=0.52; p=0.95; p=0.31, respectively) (Table 2).

All JOAG patients had bilateral disease. For the eyes in the study group, treatment was initiated with prostaglandin monotherapy for 12 eyes, prostaglandin and brimonidine for 5 eyes, and prostaglandin and brinzolamide for 5 eyes. In 3 eyes, trabeculectomy with mitomycin C was performed in order to control glaucoma.

## Discussion

JOAG, a rare form of POAG, is characterized by high IOP and glaucomatous optic disc and RNFL changes with normal ocular structure and open iridocorneal angle (±prominent iris processes) and without any systemic disorders.^1,2^ The absence of anterior segment disorders like megalocornea, buphthalmos, and other findings of anterior segment dysgenesis is the main difference between JOAG and other childhood glaucomas.^1,2^ Our hypothesis in this study was that patients with JOAG exhibit minor differences in the anterior segment. Therefore, we compared the anterior segment parameters and axial length values of JOAG patients with normal subjects. We included recently-diagnosed JOAG cases in our study and used optical biometry working with low-coherence reflectometry.^7,8^ We also compared refractive status between JOAG patients and normal subjects, as the parameters we investigated should be associated with refraction.

All of our patients had bilateral JOAG and 40% had family history of glaucoma. Our results are similar to those reported in a study by Aponte et al.^9^ in which they investigated the incidence and clinical characteristics of childhood glaucoma. In total, 13.3% of their patients had JOAG. All JOAG cases were bilateral disease and 50% of their patients had family history of the disease.^9^

Our comparison of refractive status in JOAG patients and normal subjects showed that the JOAG patients were more myopic, though the difference was not statistically significant. Park and Kee^6^ reported myopic SE values between -3.5 and -7.5 D and large diurnal variations in IOP in their JOAG patients despite maximum medical treatment. They stated that trabeculectomy was more effective in such cases to prevent glaucoma progression.^6^ Kwun et al.^2^ retrospectively investigated the clinical characteristics of 125 eyes of 72 JOAG patients. Male predominance and myopia were found to be significantly associated with JOAG in their study.^2^ We also determined that 63.6% of the JOAG patients in our study were male, consistent with previous studies. Ko et al.^10^ compared the risk factors of JOAG and POAG in their study. Myopic refractive state was significantly more common in JOAG than POAG and they stated that axial myopia might be one of the main factors in the pathogenesis of JOAG.^10^ Our findings of significantly longer AL in our JOAG patients are also supportive of Ko et al.’s^10^ results, due to the relationship between myopia and long AL.

The mean CCT values of glaucoma patients were found to be significantly lower than those of normal subjects in our study. Urban et al.^11^ investigated the CCT and endothelial cell density in adult patients with JOAG under topical anti-glaucoma treatment and compared them with ocular hypertension patients without glaucoma therapy. They found significantly lower endothelial cell density in JOAG patients but no significant differences in CCT. Unlike our study, they investigated adult patients with JOAG, as all of our patients were younger than 18 years old. Furthermore, all of our cases had recently diagnosed glaucoma and none of them had used anti-glaucoma treatment before the measurements. Tai et al.^12^ investigated CCT and corneal diameter in childhood glaucoma and found a relationship between a larger corneal diameter and thinner CCT. However, their study included patients with all types of childhood glaucoma. Also, in our study no significant differences in WTW values were found between the glaucoma and control groups. Goel et al.^13^ presented cases of keratoconus with JOAG in their study and stated that thin CCT associated with keratoconus may be an independent risk factor for glaucoma. However, none of the patients in our study had keratoconus.

JOAG patients showed deeper anterior chambers than normal subjects in this study. An inverse relationship between age and ACD has been shown in both POAG cases and normal subjects.^14^ To the best of our knowledge, there have been no previous reports that compared ACD in JOAG patients and normal subjects. This finding may be explained by the greater myopic shift of our glaucoma patients. Myopic eyes have been shown to have deeper ACD than emmetropic and hyperopic eyes.^15^ As no significant correlation between IOP and ACD has been shown among POAG cases before, the higher IOP values of our JOAG cases should not contribute to this ACD difference.^16^ No significant differences in LT, K values, or WTW values were detected in our study. We measured these parameters before antiglaucoma treatment and excluded patients with previous anti-glaucoma treatment because these agents may affect the structure of the anterior segment.^17,18^

JOAG patients show more posterior segment alterations than POAG patients. Gupta et al.^19^ compared the optic discs of primary congenital glaucoma, JOAG, and POAG cases by scanning laser ophthalmoscopy (Heidelberg Retina Tomograph III, Heidelberg Engineering, Heidelberg, Germany). They observed larger optic discs, greater horizontal C/D ratios and concentric enlargement of the cup in JOAG compared with POAG and stated that this may be related to higher IOP values.^19^ In addition, cupping reversal has been demonstrated in pediatric glaucoma cases, unlike with adults.^20,21^ The reversal of cupping was proposed to be a result of increased elasticity of the optic nerve head and lamina cribrosa in childhood.^18,19^ These posterior segment differences suggested the possibility of anterior segment alterations in juvenile glaucoma patients. Therefore, we aimed to find these differences in our study.

## Conclusion

In conclusion, we observed more myopic shift, longer AL, and thinner CCT values in JOAG compared with normal subjects in our study. To the best of our knowledge, there have been no previous reports comparing the anterior segment morphology of JOAG patients and normal subjects. The main limitation of our study is the small patient number. Further investigations with larger patient groups and different types of childhood glaucoma should be encouraged. Furthermore, a comparison with other imaging systems like rotating Scheimpflug camera system or anterior segment OCT should strengthen the results.

## Figures and Tables

**Table 1:**
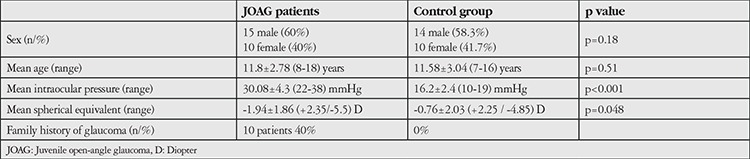
Demographic characteristics of the patients and the intraocular pressure and the spherical equivalent values of the eyes

**Table 2:**
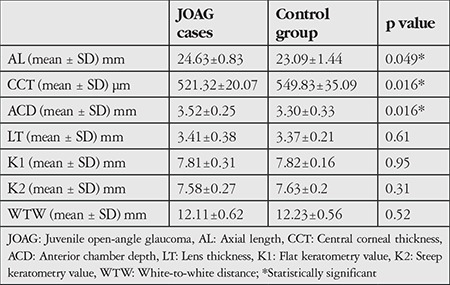
The anterior segment parameters of patients with juvenile open-angle glaucoma and control subjects
